# Characteristics and contributing factors of adverse drug reactions: an analytical study of patients with tuberculosis receiving treatment under the National TB Program of India

**DOI:** 10.12688/f1000research.125815.2

**Published:** 2024-07-23

**Authors:** Harsh Shah, Sandul Yasobant, Jay Patel, Priya Bhavsar, Somen Saha, Yogesh Patel, Deepak Saxena, Anish Sinha

**Affiliations:** 1 Department of Public Health Science, Indian Institute of Public Health, Gandhinagar, Gandhinagar, Gujarat, 382042, India; 2 School of Epidemiology and Public Health, Datta Meghe Institute of Medical Sciences, Wardha, Sawangi (Meghe), Wardha, Maharashtra, 442004, India; 3 Department of CGC Project, World Health Partners, Noida, Uttar Pradesh, 201301, India

**Keywords:** Tuberculosis, Adverse Drug Reactions, National TB Program, India

## Abstract

Background

Tuberculosis (TB) continues to pose a serious threat to the public health system in India. Although the National Tuberculosis Elimination Program (NTEP) is providing a wide range of interventions from early diagnosis to complete treatment to reduce morbidity and mortality from TB, adverse drug reactions (ADR) remain a challenge in treatment adherence and completion.

Methods

An observational cross-sectional study was conducted in selected districts of Gujarat state. A total of 593 reported TB patients were recruited with an adjusted unified distribution based on the type of cases, site of diseases, and service facility through a simple random sampling method. A semi-structured questionnaire tool was used to collect socio-demographic, clinical, and ADR-related data from the TB patients. Data was analyzed for the frequency, percentage, chi-squared, and adjusted odds ratio to find the association between the variables.

Results

The majority of the study participants were male (87.2%), aged 15 to 60 (57.8%), daily laborers (22.4%), and married (64.2%). Over 75% of individuals had pulmonary TB, with 87% having experienced their first episode, 83% being new cases, and 44.7% having a history of addiction. ADR with mild symptoms was reported by more than a quarter (29%) of TB patients during the intensive phase (77%). The association between ADR experience and drug susceptibility was significant (p<0.005) and drug-resistant TB patients experience two times more ADRs than drug-sensitive TB patients (OR 2.04). Binomial logistic regression was carried out to describe the association between various variables and occurrence of ADRs.

Conclusion

The study highlighted a need to enhance health care providers’ capacity and program structure for managing ADRs among TB patients. In order to completely eliminate TB across the country, it also emphasized the attention for a holistic and all-encompassing strategy for managing TB patients at the field level.

## 1. Introduction

Tuberculosis (TB) is a communicable disease that remains a major cause of illness and death across low and middle-income countries (LMIC) even after the discovery of novel diagnostic methods and chemotherapeutic drugs. The incidence of TB and rising numbers of multidrug-resistant TB cases are still a concern for countries with high disease burden. As per the global TB report, the incidence of TB in India is approximately 2.8 million cases annually, accounting for almost a quarter of all TB cases worldwide. Even though a six-month drug regimen can successfully treat about 85% of those who develop TB, TB remains a significant threat to public health systems due to difficulties in early detection and the required treatment duration. ^1^ Over the years, the National TB Elimination Program (NTEP) has expanded the range of anti-tuberculosis therapy (ATT) drugs utilized in daily regimens and revised programmatic guidelines for the management of drug-resistant TB. ^2^

The critical component of ATT is the standard directly observed treatment, short course (DOTS) chemotherapy regimen for drug-susceptible TB and the extended multidrug regimen for drug-resistant TB, depending on the culture and drug susceptibility tests. Poor treatment adherence increases the risk of drug resistance, treatment failures, relapses, and deaths. The persistence of infection among TB patients due to poor adherence continues to be a barrier to the success of TB programs. ^3^ To avoid morbidity, mortality, and the spread of TB, every effort should be made to persuade and motivate patients to continue their treatments despite any discomforts due to adverse drug events (ADEs). Almost all anti-TB medications result in adverse drug reactions (ADRs) that can range in severity from minor to fatal. Compared to second-line treatments, first-line anti-TB medications are often well tolerated by patients. These ADRs can cause TB patients to stop their therapy, resulting in needless morbidity, drug resistance, treatment failure, a decreased quality of life, or even death. ^4^^–^^6^ Comorbid conditions and risk factors influence the incidence of ADR and the outcome of TB treatment.

Between 8 and 85% of patients experience different side effects, ranging from mild to severe. ^5^ About 10–25% of patients who experience side effects develop significant and deadly medication reactions or serious adverse events (SAEs). ^7^^–^^9^ Treatment failure, relapse, or the formation of resistance are risks for patients who take their drugs inconsistently or stop taking them due to side effects. ^10^^–^^13^ It is crucial that all TB patients receiving therapy effectively manage and keep track of ADRs, especially major ones. Early ADR detection and prompt care can improve drug compliance, improve the treatment outcome, and stop the emergence of drug resistance. ^14^ Due to their under-recording and under-notification when monitored by the NTEP, the range and characteristics of ADR are not well recognized. With this background, the present study was conducted to assess the prevalence and characteristics of ADRs among TB patients and identify various epidemiological, socio-demographic, and programmatic factors associated with ADRs in the Western state of India, Gujarat.

## 2. Methods

### 2.1 Study design and settings

A descriptive observational cross-sectional study was conducted from 3 ^rd^ May 2021 to 30 ^th^ July 2021 in the Western state of India, Gujarat. The study was conducted through the district tB centre (DTC) and 32 tuberculosis units (TUs) in Gandhinagar and Surat districts (Gujarat state), with TB patients registered and managed. NTEP has been implemented in all districts of the state. Each district has a district TB center, which monitors the program for the entire district. The district is further divided into sub-districts i.e., TUs, at each block. Under the TUs, outlying peripheral (government and private) health facilities (PHI) provide programmatic management for TB patients.

### 2.2 Study population and sampling method

The assessment targeted a diverse profile of TB patients, such as drug-sensitive TB (DSTB), drug resistance TB (DRTB), pediatric TB, and extra-pulmonary TB. It included both public and private sector patients. The patients diagnosed with TB are reported on the online digital patient management portal Nikshay in the notification registers by the health facilities. ^15^ The list of reported TB patients from 1 ^st^ July 2018 to 31 ^st^ December 2020 was extracted from Nikshay to ensure that the study population completed treatment based on the duration of the treatment regimen. A total of 20,668 patients were reported in the Nikshay portal from both districts during that period.

The sample size was calculated based on the formula of N=Z21−α/2P(1−P)/ε2, where N=sample size, Z21−α=confidence interval, P=estimated proportion, ε=desired precision/error, with estimated proportion of 50% of ADR occurrences. Based on sample size calculation, it was derived that over 534 TB patients had to be included in the study to have a confidence level of 98% and a desired error that is within ±5% of the measured/surveyed value. Additionally, the final sample size accounted for around a ~10% non-response rate, bringing the number of study participants to about 593. The eligible TB cases were listed with the inclusion and exclusion criteria below. Inclusion criteria: the TB patients reported through Nikshay, their current state PHI was within the selected geographical areas of Gujarat state, and they were given treatment. Exclusion criteria: TB patients who migrated or were untraceable or did not reside in the current PHI surveyed areas or whose relatives didn't provide consent were excluded from the study.

From each TU, patients were recruited randomly depending on their availability and willingness to participate. Simple random sampling was adopted to select TB cases within the selected geographic areas until the saturation of the sample size. However, a proportionate adjustment based on the type of cases, service facility, and site of disease was considered for the unified distribution across the study geography to ensure the collective representation of the study participants.

### 2.3 Data variables and data collection

A semi-structured interview followed by a semi-structured, pilot-tested ADR assessment questionnaire was used to collect the data in the vernacular language (Gujarati). A pretested and semi-structured questionnaire tool consisting of information regarding primary socio-demographics, medical history, history of addiction and comorbidity, and information about the grade and type of ADRs was administered by the trained researchers in the vernacular language through personal interviews by undertaking home visits. The researchers were trained to administer the questionnaire with a participatory approach and role play to prepare them to interview the study participants for the required information.

### 2.4 Study definition for adverse drug reactions

The World Health Organization (WHO) has defined adverse drug reactions (ADRs) as “A response to a drug which is noxious and unintended, and which occurs at doses normally used in humans for the prophylaxis, diagnosis, or therapy of disease, or the modification of physiological function”. ^16^ The cornerstones of DSTB therapy continue to be a treatment plan with a minimum duration of six months and numerous first-line medicines (FLDs), such as isoniazid (H), rifampicin (R), pyrazinamide (Z), ethambutol (E), and streptomycin (S). Similar to this, NTEP offers streamlined regimens for several forms of DR-TB, including shorter oral bedaquiline-containing MDR/rifampicin resistant-TB regimens and longer oral M/XDR-TB (mono or extreme drug resistant) regimens generally ranging from six to nine months but can reach up to 20 months. The drug dosages are adjusted based on the age, weight, severity of the disease, site of the disease, and type of drug resistance/susceptibility towards ATTs.

### 2.5 Data analysis

Once the data collection was completed, data sets were scrutinized for completeness and validation by the different sets of the researchers. The study participants were contacted again if any data variables were found to be missing by the researchers who had collected the primary data. The patient data on various variables was tabulated, analyzed, and interpreted by proper statistical methods using IBM SPSS statistics software version 20 (RRID:SCR_019096). The chi-squared test was used to compare groups, while the chi-squared for the trend examined linear trends. Risk measures were determined using odds ratios (OR) and 95% confidence intervals (CI). Crude OR and 95% CI were calculated for the interpretation of univariate analysis, with the level of significance set at p<0.05. To identify the independent factors associated with ADRs, adjusted odds ratios (AOR) and 95% CI were calculated by bivariate logistic regression analysis.

## 3. Results

Based on the study criteria, 105 (18%) TB patients from Gandhinagar and 488 (82%) from Surat were included. There were 536 (90%) patients who completed the treatment and 57 (10%) on treatment.

### 3.1 Demographic profile of study participants

The mean age of study participants was 34.6±15.6 years, and the median age was 31 years, ranging from 1 to 85 years. The majority of study cases, 517 (87.2%), were in the age group of 15–60. There were 343 (57.8%) male patients and 250 (42.2 %) female patients. There were 99 (16.7%) illiterates, 52 (8.8%) graduates, and 133 (22.4%) daily laborers and 381 (64.2%) of the patients were married. The association between age categories, marital status, and education status and adverse drug reaction was not significant (p>0.05). However, among gender and occupation status, it was found to be significant (p<0.05) ( Table 1).

**Table 1.** Socio-demographic profile of the TB patients (n=593).

Socio-demographic profile of the TB patients (n=593)							
Characteristics	ADR (Yes)	(%)	ADR (No)	(%)	n	(%)	Chi-squared and p values
**Age categories**							
≤15 Years	8	1.3	23	3.9	31	5.2	Chi-squared=1.329 p value: 0.722
16 –30 years	79	13.3	184	31	263	44.4	
31 –60 Years	75	12.6	179	30.2	254	42.8	
>61 Years	10	1.7	35	5.9	45	7.6	
**Gender**							
Male	78	45.3	256	60.8	343	57.8	Chi-squared=15.505 p value: 0.0001
Female	94	54.7	156	37.1	250	42.2	
**Marital status**							
Divorce/separated/widow	5	2.9	6	1.4	11	2.0	Chi-squared=5.407 p value: 0.067
Married	99	57.6	282	67.0	381	64.2	
Single	68	39.5	133	31.6	201	33.8	
**Education status**							
Illiterate	30	17.4	69	16.4	99	16.7	Chi-squared=2.466 p value: 0.651
Primary	56	32.6	165	39.2	221	37.3	
Secondary	43	25.0	96	22.8	139	23.4	
Higher secondary	27	15.7	55	13.1	82	13.8	
Graduate and above	16	9.3	36	8.6	52	8.8	
**Occupational status**							
Daily labourer/farmer/cultivator	28	16.3	147	34.9	175	29.5	Chi-squared=24.5266 p value: 0.0001
Employed	30	17.4	74	17.6	104	17.5	
Housewife	56	32.6	85	20.2	141	23.8	
Business or professional	42	24.4	77	18.3	119	20.1	
Student	16	9.3	38	9.0	54	9.1	

### 3.2 Clinical profile of the study participants

The study participants were comprised of 147 (25%) extra-pulmonary TB (EPTB) patients and 446 (75%) pulmonary TB patients (PTB), 519 (87%) of whom had contracted the first episode of TB. The distribution of the type of cases as per national guidelines was 492 (83%) new cases and 69 (12%) previously treated cases on the drug-sensitive TB treatment regimen, while 32 (5%) cases were on the drug-resistant treatment regimen. A total of 66 (11%) TB patients were receiving treatment from private providers. The study reported that 268 (44.7%) had a history of addiction, with 91% addicted to tobacco (either smokeless or smoking) and 9% to alcohol. Among them, 41% had an addiction to tobacco and alcohol, while 1.2% had addictions to psychotic substances. Sixty-one (10%) reported the presence of at least one comorbidity, while the major contribution of comorbidity was diabetes (50%). The number of patients with a HIV co-infection was deficient in numbers to be included in a detailed analysis.

### 3.3 Adverse drug reactions among the study participants

During the study, it was observed that 172 (29%) patients experienced ADRs with at least one symptom. Out of those, 80% had mild symptoms, and 133 (77%) experienced them during the early (intensive) phase of the treatment initiation. The 18 (56%) drug-resistant TB patients on second-line ATTs reported ADR, 50% of whom reported moderate and severe ADRs. The association between ADR experience and drug susceptibility was significant (p value of 0.005; Chi-squared 12.193) and drug-resistant TB patients experience two times more ADRs than drug-sensitive TB patients (OR 2.049, CI: 1.47–2.86). The TB patients had experienced gastric disturbances, skin-related symptoms, peripheral nervous system symptoms, arthralgia, ophthalmic discomfort, and psychological disorders during ATT ( Table 2).

**Table 2.** Symptoms of adverse drug reactions in TB patients (n=172) during anti-tuberculosis therapy (multiple answers).

Symptoms of adverse drug reactions	n	%
Gastric discomfort (nausea/vomiting/gastric discomfort)	97	56.4
Skin related reactions (itching/redness)	59	34.3
Peripheral nervous system (numbness/tingling)	37	21.5
Joint pain (arthralgia/joint stiffness)	76	44.2
Ophthalmic discomfort (impaired vision/red eyes)	27	15.7
Psychological disturbances (confusion/anxiety)	18	10.5

### 3.4 Logistic regression on predictive independent variables

The study used a binomial logistic regression model to estimate the bivariate odds ratio and a 95% confidence interval to describe the association between predictor variables and the occurrence of ADRs. The study used the dataset's socio-demographic, clinical, and programmatic service delivery variables to develop the predictive model. The model showed that gender, drug susceptibility status, and history of addiction were statistically significant (p<0.05). The regression model showed that the Nagelkerke R2 value was 0.139 with a classification accuracy of 71% ( Table 3).

**Table 3.** Logistic regression on predictive independent variables: socio-demographic, clinical and programmatic services of adverse drug reactions (n = 593).

		ADR experienced				Total	%	B	Wald	aOR	(95% CI)	p value
		No	%	Yes	%							
Gender	Female	156	26.3	94	15.9	250	42.2	0.883	19.187	2.419	(1.629–3.592)	0.000
	Male	265	44.7	78	13.2	343	57.8			Reference		
Literacy	Illiterate	69	11.6	30	5.1	99	16.7	0.021	0.006	1.021	(0.612–1.704)	0.937
	Literate	352	59.4	142	23.9	494	83.3			Reference		
Anatomical site	Extra-pulmonary TB	101	24.0	46	26.7	147	24.8	0.119	0.281	1.126	(0.726–1.748)	0.596
	Pulmonary TB	320	76.0	126	73.3	446	75.2			Reference		
Drug susceptibility	DRTB	14	2.4	18	3.0	32	5.4	1.523	14.158	4.587	(2.075–10.141)	0.000
	DSTB	407	68.6	154	26.0	561	94.6			Reference		
Service facility current	Private providers	46	7.8	20	3.4	66	11.1	0.190	0.389	1.209	(0.0665–2.198)	0.533
	Public health facility	375	63.2	152	25.6	527	88.9			Reference		
Episodes of TB	More than one episode	56	9.4	18	3.0	74	12.5	0.528	2.730	1.696	(0.906–3.175)	0.098
	First episode	365	61.6	154	26.0	519	87.5			Reference		
History of addiction	Yes	164	27.7	101	17.0	265	44.7	1.138	30.224	3.121	(2.080–4.684)	0.000
	No	257	43.3	71	12.0	328	55.3			Reference		
History of comorbidity	Yes	42	7.1	19	3.2	61	10.3	0.260	0.630	1.297	(0.682–2.466)	0.427
	No	379	63.9	153	25.8	532	89.7			Reference		
Age (Years)								−0.008	1.285	0.992	(0.978–1.006)	0.257
Constant								−2.220	27.510	0.109		0.000

Number in () indicates the row % across the group and column % in total.

All variables shown here significantly differ across the group with p<0.001.

Cox & Snell R Square 0.097, Nagelkerke R ^2^ value was 0.139, classification accuracy is of 71% in prediction.

## 4. Discussion

Adverse events, defined as any unfavorable medical occurrence, can also be linked to treatment with these medications but are not always causally related. The study was conducted in only a selected part of the country, but the findings of the study provide insight into the drug reactions observed by TB patients during the course of treatment. The present study revealed that the prevalence of ADRs was 29% among the study population, similar to various worldwide studies ranging from 8% to 85%. ^3^^–^^6^^,^^14^^,^^17^^–^^20^ Several studies reported more ADR prevalence in drug-resistant TB patients, similar to the present study, where 50% of DRTB patients experienced ADRs. ^19^^,^^21^^–^^23^ The variance in ADR prevalence between these studies could be due to several data collection variables including the ADR reporting mechanism, patient-reported (subjective) or clinician-detected (objective), and variations in the use of particular anti-tubercular drugs such as dosage and ancillary medications used for ADR management.

The study observed 71% mild grade ADRs, 77% of which occurred in the early period (intensive phase) of treatment. Several studies also reported that major or severe ADRs were less common (occurring in approximately 2% of the cases, reaching 8% in specialized clinics), and ADRs were more prevalent in the intensive phase than in the continuation phase. ^24^^–^^27^ Many studies have reported the frequency of symptoms and types of ADRs, which can range in severity from mild to severe, caused by both first-line and second-line anti-TB medications. The drug-specific ADRs may cause either a reduction of dosage or termination of the offending drug(s) and lead to common ADRs up to organ-specific toxicity in severe cases. ^3^^,^^21^ The present study reported that the most common ADRs were gastric discomfort and arthralgia, followed by cutaneous ADRs, peripheral neuropathy, ophthalmic photosensitivity, and psychiatric disorders (headache/anxiety/confusion), similar to various studies conducted in India. ^22^^,^^28^^–^^30^ When compared to other adverse events, patients report gastrointestinal adverse events and arthralgia more frequently, which can contribute to subjective variation and a high prevalence of these events. Similar findings were observed in a study that showed gastrointestinal symptoms as the most common among persons with TB treated with DSTB regimen, followed by arthralgia, cutaneous drug reactions, and peripheral neuropathy. Gastrointestinal reactions are likely due to the oral administration of medications. These findings highlight the common adverse effects of TB medications and emphasize the need for effective management strategies to improve patient outcomes. ^31^

A study from Uttar Pradesh by Prasad *et al*. and Gujarat by Jakasania *et al*. reported that there was no statistically significant difference in patients suffering from ADRs concerning variables such as age group, gender, educational and occupational status, history of addiction, and presence of a comorbidity, episodes of TB, and healthcare facility opted for services. ^19^^,^^29^ According to the study, one of the associated factors for ADR is the female gender. However, we may not have observed this since most participants in our study cohort were men. The logistic regression model of the present study identified that gender, drug susceptibility, and history of addiction were each predisposing risk factors for ADRs.

### Limitations

The present study recorded ADRs or adverse events from the history of patients that could lead to subjective variations and recall bias. The type and grade of ADRs were also recorded from the patient's perspective, limiting the researchers to identify the drug-specific symptom of ADRs. In the absence of patients' medical records, the study could not assess or record the nutritional status at the time of ATT, the severity of comorbidity, drug specific ADRs or confirm hospitalisation due to severe ADRs. This was one of the reasons that during the study, researchers had not considered the history of stoppage of the offending drug(s), alterations in the treatment regimen, or management received for the discomfort. The study excluded one TB patient who was non-traceable at the time of the data collection. The excluded TB patient could have provided additional information that support the results.

## 5. Conclusions

The present study focused on adverse events pertaining to TB patients missed by the health system. The analysis delivered crucial conclusions that could direct policymakers to educate and train all healthcare professionals and high-risk patients on how to solicit and manage ADRs among patients receiving programmatic treatment effectively. It is crucial to strengthen the program by carefully examining treatment plans based on medical history, ensuring treatment compliance, managing adverse events aggressively and proactively, and establishing a training cascade for health care providers and treatment supporters.

## Ethics and informed consent

Ethical approval for this study was obtained from the Indian Institute of Public Health Gandhinagar- Institutional Ethics Committee (TRC-IEC No:18/2020-21). The administrative approval to conduct the study was received from the State TB cell, Department of Health and Family Welfare, Government of Gujarat.

Written informed consent for publication of the patients’ details was obtained from the patient or the parents/guardian of the participant if they were under 18 years of age.

## Author contributions

All authors contributed equally to the development of this study. All authors contributed to data analysis, drafting, or revising the article, have agreed on the journal to which the article will be submitted, gave final approval of the version to be published, and agreed to be accountable for all aspects of the work.

## Data Availability

Figshare: A Study on Adverse Drug Reaction Among TB Patients, https://doi.org/10.6084/m9.figshare.21185875.v1. ^32^ The project contains the following underlying data:
- Gujarat Baseline Data for Online Publication.xlsx - Protocol Methodology for the study assessing the Adverse Drug Reactions among TB patients.docx - ADR Questionnaire Tool - Gujarati.docx (in the language in which the questionnaire was distributed) - ADR Questionnaire Tool.docx (in English) Figshare: STROBE checklist for ‘Characteristics and contributing factors of adverse drug reactions: an analytical study of patients with tuberculosis receiving treatment under the National TB Program of India’, https://doi.org/10.6084/m9.figshare.21185875.v1. ^32^ Data are available under the terms of the Creative Commons Attribution 4.0 International license (CC-BY 4.0).
