# Supplementary material for: Characteristics and contributing factors of adverse drug reactions: an analytical study of patients with tuberculosis receiving treatment under the National TB Program of India
Source: F1000Res. 2024 Jul 23;11:1388. Originally published 2022 Nov 25. [Version 2] doi: 10.12688/f1000research.125815.2 (PMC11811607; doi:10.12688/f1000research.125815.2)
Supplement: Peer review history

# Reviewer report: Reviewer response for version 2

**DOI:** 10.5256/f1000research.169379.r361948

**Published:** 2025-02-07

**Authors:** Anurag Agarwal

**Affiliations:** 1 Maulana Azad Medical College & Associated Hospitals, New Delhi, India

This study was conducted to assess the prevalence and characteristics of ADRs among TB patients and identify various epidemiological, socio-demographic, and programmatic factors associated with ADRs in the Western state of India, Gujarat.

  I have the following observations:

 1. First line ATT and 2nd line ATT adverse effects are not comparable and should be handled separately. This is so as drugs, duration of treatment and number of drugs too are different and not comparable.

 2. A comparison of ADRs within weight bands could have been made. That is, patients lying in which part of weight band had more adverse events. Was there any relation to weight in the individual weight band?

 3. Was there an increase in adverse events after shifting to daily ATT vs previous alternate day ATT?

 4. Hospitalization and temporary or permanent drug stoppage, mortality should have been recorded.

 5. How was it concluded that " The analysis delivered crucial conclusions that could direct policymakers to educate and train all healthcare professionals and high-risk patients on how to solicit and manage ADRs among patients receiving programmatic treatment effectively. It is crucial to strengthen the program by carefully examining treatment plans based on medical history, ensuring treatment compliance, managing adverse events aggressively and proactively, and establishing a training cascade for health care providers and treatment supporters."

 This was not the aim or purpose of the study nor was it looked at in the study.

Is the work clearly and accurately presented and does it cite the current literature?

Yes

If applicable, is the statistical analysis and its interpretation appropriate?

Yes

Are all the source data underlying the results available to ensure full reproducibility?

Yes

Is the study design appropriate and is the work technically sound?

Yes

Are the conclusions drawn adequately supported by the results?

No

Are sufficient details of methods and analysis provided to allow replication by others?

Yes

Reviewer Expertise:

Infectious diseases, rabies, tuberculosis

I confirm that I have read this submission and believe that I have an appropriate level of expertise to confirm that it is of an acceptable scientific standard, however I have significant reservations, as outlined above.

# Reviewer report: Reviewer response for version 2

**DOI:** 10.5256/f1000research.169379.r306199

**Published:** 2024-08-19

**Authors:** Gyanshankar Mishra

**Affiliations:** 1 Indira Gandhi Government Medical College, Nagpur, Maharashtra, India

With the revisions incorporated, the article now offers a comprehensive overview, discussing key points and proposing potential policy recommendations for diagnosing and managing adverse drug reactions (ADRs)  to anti TB drugs in programmatic settings.

Is the work clearly and accurately presented and does it cite the current literature?

Partly

If applicable, is the statistical analysis and its interpretation appropriate?

I cannot comment. A qualified statistician is required.

Are all the source data underlying the results available to ensure full reproducibility?

Yes

Is the study design appropriate and is the work technically sound?

Yes

Are the conclusions drawn adequately supported by the results?

Yes

Are sufficient details of methods and analysis provided to allow replication by others?

Yes

Reviewer Expertise:

Respiratory diseases, Tuberculosis

I confirm that I have read this submission and believe that I have an appropriate level of expertise to confirm that it is of an acceptable scientific standard.

# Reviewer report: Reviewer response for version 2

**DOI:** 10.5256/f1000research.169379.r305305

**Published:** 2024-07-25

**Authors:** Aditi Gupta

**Affiliations:** 1 Pulmonary Medicine, BRAIMS, Mohali, India

Previous suggestions are not being considered.

Is the work clearly and accurately presented and does it cite the current literature?

Yes

If applicable, is the statistical analysis and its interpretation appropriate?

Yes

Are all the source data underlying the results available to ensure full reproducibility?

Yes

Is the study design appropriate and is the work technically sound?

Partly

Are the conclusions drawn adequately supported by the results?

Yes

Are sufficient details of methods and analysis provided to allow replication by others?

Partly

Reviewer Expertise:

NA

I confirm that I have read this submission and believe that I have an appropriate level of expertise to state that I do not consider it to be of an acceptable scientific standard, for reasons outlined above.

# Response

**Published:** 2024-07-25

**Authors:** Harsh Shah

**Affiliations:** Public Health Science, Indian Institute of Public Health, Gandhinagar, Gandhinagar, Gujarat, India

Greetings, Reviewer

 We appreciate your input, however we would like you to review the response that addresses the comments you expressed regarding the manuscript. We believe that the evidence that this article generates will be necessary to improve India's ADR surveillance system as part of the National TB Elimination Program.

 1. Kindly elaborate Serious adverse events (SAE) and ADR categories also need to be explained

- The study had classified ADRs into various categories based on severity, including mild, moderate, and severe, with SAEs defined as those resulting in significant health risks, requiring hospitalization, or leading to long-term consequences. This classification is based on the guidelines and protocols defined in context with the National TB Elimination Program (NTEP).

2. It has been proven that elderly develop more ADR but this is not the case with your study.

- While literature indicates a higher prevalence of ADRs among elderly patients, the findings may reflect demographic variances or differences in treatment regimens that could mitigate these risks. It is crucial to consider that the elderly in the study may have had different TB comorbidities or medication profiles compared to the general population. The results provide valuable insights into the complexity of ADRs and underscore the need for targeted interventions in vulnerable groups.

3. BMI, age, comorbidity have strong association with ADR. Given tables and text do not

 support this.

- While the study did not establish significant correlations in the reported data, it is important to consider that these associations can vary based on geographical and socioeconomic factors affecting the patient population. The findings contribute to the broader understanding of ADRs in TB treatment and highlight the necessity for ongoing research to explore these relationships further, particularly in public health settings where such data can inform resource allocation and management strategies. We made references supporting this evidences, however we believe there should be more in depth operational research or longitudinal case control study will provide more structured findings in alignment of the NTEP in India. 

4. Kindly elaborate, how did you reach private patients for interview.

- We reached out the private patients through the National Tuberculosis Elimination Program (NTEP) staff, project staff and through various stakeholders, to ensure a comprehensive representation of persons with TB across both public and private healthcare sectors.

5. Since, there is lot of recall bias, results cannot be generalized.

- While we acknowledge that recall bias can affect data accuracy, we have already mentioned and narrated it under the limitation of the study. In order to reduce the recall bias, we had further employed a semi-structured questionnaire designed to mitigate these issues by prompting patients to recall their experiences in the context of their ongoing / completed treatment. We ensured that the interviewed study subjects have the medical records available in Ni-kshay or with treatment report card at health facilities in case of requirement to check bias.   

6. At what time of treatment, did you interview the patient? Time of interview is very

 important in assessing ADR and decreasing recall bias. Ideally different groups at different

 time point of treatment must be evaluated or even same group at different time points if

 feasible.

- The study interviews were conducted from the individuals with TB on post completion / on going of their TB treatment. We have tried to incorporate various representative of patients in the study to understand the occurrence of ADRs at various phase of treatment. We could not make separate groups in absence of budgetary restriction to repeat visits for periodic timeframe. The study design was cross sectional and interview was one time event. This is limitation that we have mentioned into the manuscript.

7. Design of the study makes it very less informative.

- While the design may appear limited, it was intentionally developed to capture data from a diverse patient population. The findings provide critical insights into ADRs experienced by individuals undergoing TB treatment at point interval (with cross sectional design), which is vital for program policies aimed at improving drug safety and efficacy.
- The study highlights the importance of monitoring ADRs during various treatment phases, which is essential for developing effective TB interventions that ensure patient safety and optimize treatment outcomes.

Kindly do revisit the manuscript and comments that we addressed here. 

 Regards

# Reviewer report: Reviewer response for version 1

**DOI:** 10.5256/f1000research.138161.r235640

**Published:** 2025-02-10

**Authors:** Yogendra Shah

**Affiliations:** 1 Province Public Health Laboratory, Kailali, Nepal; 2 Infectious Disease, National One Health Alliance for Nepal, Kathandu, Bagmati, Nepal

Title: Characteristics and Contributing Factors of Adverse Drug Reactions: An Analytical Study of Patients with Tuberculosis Receiving Treatment under the National TB Program of India

 Reviewer: I would like to suggest some improvements to enhance the manuscript's acceptability. Please find below point-wise comments to refine the manuscript:

 Title: Revise the title to align more closely with the manuscript's research objectives.

 Abstract: Summarize only the significant findings of the manuscript, including background, rationale, and justification of the study. Include information on research objectives, methods, findings, and recommendations.

 Keywords: Select crucial keywords from the abstract.

 Introduction:

- Provide a brief overview of the global situation concerning *Mycobacterium tuberculosis* complex, with a specific focus on the current status in India.
- Discuss the utility of phenotypic and genotypic techniques in diagnosing TB globally. Address questions such as the prevalence of MDR and pan-susceptible strains, predominant lineages among MDR and non-MDR strains, mechanisms of drug resistance, and the challenges of detecting transmission dynamics without epidemiological data.
- Explore possible contributing factors to adverse drug reactions among tuberculosis patients. Justify the feasibility of combining epidemiological, phenotypic, genotypic, and WGS data for spatial analysis of TB transmission dynamics.
- Examine drug-resistant genes' mechanisms through genotypic techniques, explaining principles and applications for diagnosing TB and other diseases.
- Clearly justify the research objective and rationale for the current study.

Materials and Methods:

- Define inclusion and exclusion criteria for confirming TB patients using phenotypic and genotypic methods.
- Concisely outline procedures for phenotypic methods (sputum collection, microscopic examination, GeneXpert, culture) and genotypic methods. Consider creating a flowchart illustrating the study design and procedures.
- Provide a straightforward discussion of procedures and statistical analyses in an easily understandable format.
- Elaborate on the procedure for studying drug-resistant TB isolates and the use of artificial intelligence software for vaccine development against TB.

Results:

- Restructure and discuss results in a sequential order for better comprehension.
- Compare various databases for studying mutations in resistance genes to first and second-line anti-TB drugs.
- Highlight key findings in tables and provide brief explanations. Use asterisks to denote P<0.05 significance in tables.

Discussion:

- Revise the entire discussion section, comparing the current paper's findings with those of previous global studies. Offer logical explanations to enhance understanding.
- Include a discussion on the limitations of the research study.
- Conclude with comprehensive recommendations grounded in practicality rather than theory.
- Implementing the current study involves carefully following the research plan outlined in your manuscript. Here is a step-by-step guide on how to implement the study:

Is the work clearly and accurately presented and does it cite the current literature?

Partly

If applicable, is the statistical analysis and its interpretation appropriate?

Partly

Are all the source data underlying the results available to ensure full reproducibility?

Yes

Is the study design appropriate and is the work technically sound?

Partly

Are the conclusions drawn adequately supported by the results?

Partly

Are sufficient details of methods and analysis provided to allow replication by others?

Yes

Reviewer Expertise:

Infectious diseases with Zonootic and vector borne diseases

I confirm that I have read this submission and believe that I have an appropriate level of expertise to confirm that it is of an acceptable scientific standard, however I have significant reservations, as outlined above.

# Reviewer report: Reviewer response for version 1

**DOI:** 10.5256/f1000research.138161.r301300

**Published:** 2024-07-23

**Authors:** Aditi Gupta

**Affiliations:** 1 Pulmonary Medicine, BRAIMS, Mohali, India

1. Kindly elaborate Serious adverse events (SAE) and ADR categories also need to be explained
2. It has been proven that elderly develop more ADR but this is not the case with your study.
3. BMI, age, comorbidity have strong association with ADR. Given tables and text do not support this.
4. Kindly elaborate, how did you reach private patients for interview.
5. Since, there is lot of recall bias, results cannot be generalized.
6. At what time of treatment, did you interview the patient? Time of interview is very important in assessing ADR and decreasing recall bias. Ideally different groups at different time point of treatment must be evaluated or even same group at different time points if feasible.
7. Design of the study makes it very less informative.

Is the work clearly and accurately presented and does it cite the current literature?

Yes

If applicable, is the statistical analysis and its interpretation appropriate?

Yes

Are all the source data underlying the results available to ensure full reproducibility?

Yes

Is the study design appropriate and is the work technically sound?

Partly

Are the conclusions drawn adequately supported by the results?

Yes

Are sufficient details of methods and analysis provided to allow replication by others?

Partly

Reviewer Expertise:

NA

I confirm that I have read this submission and believe that I have an appropriate level of expertise to state that I do not consider it to be of an acceptable scientific standard, for reasons outlined above.

# Response

**Published:** 2024-07-25

**Authors:** Harsh Shah

**Affiliations:** Public Health Science, Indian Institute of Public Health, Gandhinagar, Gandhinagar, Gujarat, India

Dear Reviewer, 

 We request you to approve this article as we have incorporated your suggestions in our manuscript. Kindly do needful for its wider dissemination. 

 Regards

# Reviewer report: Reviewer response for version 1

**DOI:** 10.5256/f1000research.138161.r235642

**Published:** 2024-02-27

**Authors:** Gyanshankar Mishra

**Affiliations:** 1 Indira Gandhi Government Medical College, Nagpur, Maharashtra, India

**"** This manuscript provides a thorough analysis of adverse drug reactions (ADRs) associated with first and second line anti-tuberculosis (TB) treatment drugs. It offers valuable insights into the ADRs experienced by patients in the National TB Program of India, making a significant contribution to the existing body of literature.

 One key finding is the increased incidence of ADRs in patients with drug-resistant TB compared to those with drug-sensitive TB. This observation is crucial for a deeper understanding of the challenges in treating drug-resistant TB. I recommend enhancing the discussion on this point at the end of the first paragraph following the line addressing the variance in ADR prevalence. It would be pertinent to include a statement that emphasizes the doubling of ADRs in patients with drug-resistant TB compared to those with drug-sensitive TB, reflecting the lesser efficacy and increased toxicity of second-line drugs. This insight underscores the necessity for prolonged treatment durations and a higher expectation of ADRs during the course of second-line drug therapy.

 Additionally, the study's identification of the most common ADRs, including gastric discomfort, arthralgia, and cutaneous reactions, is consistent with findings from other studies in India. In the discussion section following the mention of these ADRs, I suggest incorporating a reference to a significant study involving 750 TB patients who received the daily fixed-dose combination anti-TB treatment under the National TB Elimination Program (NTEP) [1]. This study provides important context on peripheral neuropathy, particularly in drug-resistant TB cases treated with second-line drugs. Emphasizing the importance of early recognition and management of linezolid-associated peripheral neuropathy to prevent irreversible progression would be valuable [2]. Furthermore, the role of arthralgia, especially in relation to pyrazinamide use, warrants additional discussion. Highlighting the importance of serum uric acid estimation in differentiating hyperuricemic from normouricemic arthralgia and the potential role of uric acid-lowering drugs, in addition to standard therapies, in managing anti-TB treatment-induced hyperuricemic arthritis, is crucial for a comprehensive understanding of these ADRs [1].

 The inclusion of these points will enhance the depth and comprehensiveness of the discussion in the manuscript, ensuring it provides a detailed and nuanced understanding of the ADRs associated with TB treatment in India. The manuscript, with these additions, will be an invaluable resource for healthcare professionals and researchers in the field of TB treatment.

Is the work clearly and accurately presented and does it cite the current literature?

Partly

If applicable, is the statistical analysis and its interpretation appropriate?

I cannot comment. A qualified statistician is required.

Are all the source data underlying the results available to ensure full reproducibility?

Yes

Is the study design appropriate and is the work technically sound?

Yes

Are the conclusions drawn adequately supported by the results?

Yes

Are sufficient details of methods and analysis provided to allow replication by others?

Yes

Reviewer Expertise:

Respiratory diseases, Tuberculosis

I confirm that I have read this submission and believe that I have an appropriate level of expertise to confirm that it is of an acceptable scientific standard, however I have significant reservations, as outlined above.

## References

[1] : Adverse Drug Reactions to a Daily Fixed-dose Combination Based Antituberculosis Treatment Regime in India’s National Tuberculosis Elimination Programme: A Prospective Cohort Study. *JOURNAL OF CLINICAL AND DIAGNOSTIC RESEARCH* .2022; 10.7860/JCDR/2022/57878.16719 10.7860/JCDR/2022/57878.16719

[2] : Adverse drug reactions due to linezolid in the programmatic management of drug-resistant tuberculosis in India: A retrospective multicenter study. *Indian Journal of Tuberculosis* .2023; 10.1016/j.ijtb.2023.04.006 10.1016/j.ijtb.2023.04.006 39067941

# Response

**Published:** 2024-07-18

**Authors:** Harsh Shah

**Affiliations:** Public Health Science, Indian Institute of Public Health, Gandhinagar, Gandhinagar, Gujarat, India

Dear [Reviewer/Editor],

 The revised manuscript has incorporated the feedback from the peer review process. The suggestions from the mentioned study have been integrated into the discussion sections.

 One limitation of the study was that drug-specific adverse drug reactions (ADRs) were not established, and general considerations of ADRs were taken into account, particularly in drug-resistant tuberculosis (DR-TB) cases, due to the absence of specific case records. Therefore, linezolid-specific ADRs or those related to second-line drugs were not explored, as the primary intention was to identify general characteristics and contributing factors.

 However, the feedback received highlights the necessity for further exploration to address the continuum of treatment by creating a system that offers more detailed guidance on ADRs. In the same direction, the present study underscores the need to equip frontline workers and medical officers in health facilities with the knowledge and skills to manage ADRs efficiently.

 Thank you for your valuable insights and suggestions, which have significantly contributed to improving the manuscript.
